# MDM2 inhibition is synthetic lethal with PTEN loss in colorectal cancer cells via the p53-dependent mechanism

**DOI:** 10.7150/ijbs.82566

**Published:** 2023-07-09

**Authors:** Guowen Ren, Eun Ju Yang, Shishi Tao, Pui Kei Mou, Yue Pu, Li-Jie Chen, Joong Sup Shim

**Affiliations:** 1 Cancer Centre, Faculty of Health Sciences, University of Macau, Avenida da Universidade, Taipa, Macau SAR, China.; 2 MOE Frontiers Science Centre for Precision Oncology, University of Macau, Taipa, Macau SAR, China.

**Keywords:** PTEN, colorectal cancer, MDM2, p53, synthetic lethality

## Abstract

Colorectal cancer (CRC) driven by *PTEN* deficiency exhibits high risk of metastasis, advancement of tumor stages and chemotherapy resistance, where no effective therapy has been developed. In this study, we performed a synthetic lethal drug screening in CRC and found that PTEN-deficient CRC cells are highly vulnerable to MDM2 inhibition. MDM2 inhibitor treatment or its silencing selectively inhibited the growth of PTEN-deficient CRC in vitro and in mice models. Mechanistically, PTEN loss increased the level of active AKT and subsequently increased MDM2 phosphorylation, thereby limiting the p53 functions in *PTEN*^-/-^ CRC cells. MDM2 inhibition in turn activated p53 in CRC, particularly in *PTEN*^-/-^ CRC cells. The synthetic lethal effect of MDM2 inhibitor was largely dependent on p53, because p53 silenced cells or cells lacking p53 failed to exhibit synthetic lethality in PTEN-deficient cells. We further showed that MDM2 inhibition led to the p53-dependent reversal of Bcl2-Bax ratio, which contributed to mitochondria-mediated apoptotic cell death in PTEN-deficient CRC. This study suggests that pharmacological targeting of MDM2 could be a potential therapeutic strategy for PTEN-deficient CRC.

## Introduction

Colorectal cancer (CRC) is the one of most common cancers worldwide, showing an increasing tendency on morbidity and deaths. Various genetic alterations drive the initiation and progression of CRC, including mutational loss or inactivation in tumor suppressors such as APC, TP53, SMAD4 and PTEN 1. Among the tumor suppressors, phosphatase and tensin homolog (PTEN) is associated with advanced cancer stage and poor prognosis 2. PTEN mutation frequently occurs in CRC with distant metastasis, which contributes to 40% of sporadic CRC patients' death. Around 18~30% of sporadic CRC harbor PTEN mutation 3, 4, and 75% PTEN alteration was observed in aggressive CRC 5, 6. Moreover, colorectal tumors with PTEN inactivation are shown to be accompanied with the resistance to the chemotherapies 7, 8. These data indicated the critical roles of PTEN tumor suppressor in CRC progression, therapy responses and the high rate of mortality.

PTEN is a dual protein and lipid phosphatase located on chromosome 10. The canonical function of PTEN is to dephosphorylate phosphatidylinositol (3,4,5)-triphosphate (PIP3) to generate PIP2 and inhibit PI3K/AKT pathway, a major pro-survival and growth pathway in cancer cells 9. PTEN loss leads to persistent activation of the PI3K/AKT axis and promotes tumor growth and survival 10. Therefore, inhibition of PI3K/AKT kinases has been considered as a standard approach for the treatment of tumor with PTEN loss 11. However, most of kinase inhibitors targeting PI3K/AKT resulted in poor clinical responses in clinical trials. Moreover, inhibiting PI3K/AKT axis often generated the activation of parallel signaling or alternative survival pathways, resulting in the emergence of tumor drug resistance. Hence, there has been an urgent agenda to find alternative strategies to tackle tumors with PTEN loss.

Synthetic lethality, which arises when two genes have a genetic interaction where a cancer cell can survive under a deficiency in one gene but not in both, is one of very few possible approaches to target tumor suppressor mutations in cancer 12. It enables pharmacologically target mutant tumor suppressors with high cancer cell specificity, thus being widely investigated in cancer targeted therapy. To target PTEN loss in CRC, we carried out a synthetic lethal drug screening with PTEN-isogenic CRC cell pairs in a highly target-selective small molecule drug library. The screening identified MDM2 as a potential actionable target in PTEN-deficient CRC. MDM2 is the major negative regulator of p53 by promoting the nuclear export and degradation of p53 13. The present study shows that MDM2 inhibition is synthetic lethal with PTEN loss in CRC and the synthetic lethality is mediated by p53 and its transcriptional targets in PTEN-deficient CRC. Our study provides a novel therapeutic strategy for the treatment of CRC with PTEN loss.

## Materials and Methods

### Cell lines and culture

The three CRC cell lines, including HCT116, RKO and DLD1 were acquired from American Type Culture Collection (ATCC). *PTEN^-/-^* cell lines of HCT116, RKO and DLD1 were generated by CRISPR-Cas9 gene editing system based on the method described previously 7. The HCT116 and DLD1 PTEN*-*isogenic cell pairs were cultured in Gibco Roswell Park Memorial Institute (RPMI) 1640 medium (Thermo Fisher Scientific, Waltham, MA) with 10% fetal bovine serum (FBS). The RKO PTEN-isogenic cell pair was cultured in Gibco Dulbecco's Modified Eagle Medium (DMEM) supplemented with 10% FBS. All the cell lines are cultured under 37 °C with 5% CO2 in humidified incubator.

### Highly Selective Inhibitor Library screening

Highly Selective Inhibitor Library (384-well-L3500) was purchased from Selleck Chemical, which contains 318 small molecule inhibitors selectively targeting various cellular druggable targets. The drug library was prepared in an 8-dose interplate dilution format in 384-well plates with the concentration range from 45 nM to 100 μM. HCT116 *PTEN^+/+^* and* PTEN^-/-^* cells (1,000 cells/well) were screened in parallel with the same drug library sets. Cells were incubated in 37°C incubator for 96 h and the cell viability was measured with AlamarBlue assay. The screening was repeated twice. The IC_50_ value of each compound for each isogenic cell line was calculated by GraphPad Prism 6 (GraphPad Software, La Jolla, CA). The potential synthetic lethality candidates were selected based on the selectivity index (SI = IC_50_-HCT116 *PTEN^+/+^*/IC_50_-HCT116* PTEN^-/-^*). The compounds with SI>2.5 were considered as synthetic lethality candidates.

### Cell viability assay

Cells were seeded in a 96-well plate at a density of 5 × 10^3^ cells/well. Drug treatments were performed after cells adhering for 24 h. The cell viability was measured by AlamarBlue reagent (Thermo Fisher Scientific). AlamarBlue solution containing 0.025% (w/v) resazurin sodium salt (Sigma) was dissolved in sterile PBS (Gibco). 10% AlamarBlue solution was added to the cell culture medium and incubated at 37°C. The AlamarBlue fluorescence was measured with a SpectraMax M5 fluorescence microplate reader at Ex560/Em590 (Molecular Devices, Sunnyvale, CA). The cell images were collected by an IncuCyte ZOOM (Essen BioScience, Ann Arbor, MI).

### Cell cycle and apoptosis assays

For cell cycle analysis, cells treated with CGM097 were digested and collected, washed with PBS and fixed using precool ethanol (Sigma) at -20°C overnight. The fixed cells were washed with PBS and stained using PBS with 100 μg/ml Ribonuclease A (RNase A), 50 μg/ml propidium iodide (PI) and 0.1% Triton X-100 at 4°C overnight. Raw data were collected via BD Accuri C6 flow cytometer (BD Biosciences, CA) and analyzed via FlowJo software.

For apoptosis analysis, Cells were stain and detected by Alexa Fluor 488 annexin V/Dead Cell Apoptosis Kit (Thermo Fisher Scientific) according to the manufacturer's instruction. Raw data were collected via BD Accuri C6 flow cytometer and analyzed via FlowJo software.

### Measurement of mitochondrial membrane potential

Mitochondrial membrane potential in cells was measured with JC-1 staining kit (C2006, Beyotime, China). In brief, cells were incubated with JC-1 staining buffer for 15 min at 37°C, then observed under a Zeiss LSM 880 Confocal microscope System (Carl Zeiss, Thornwood, NY). Carbonyl cyanide m-chlorophenylhydrazone (CCCP, 10 µM) was used as a positive control for inhibiting mitochondrial membrane potential.

### Western blot analysis and antibodies

Whole cell proteins were extracted from cells with a 2X Laemmli sample buffer, which prepared by 65.8 mM pH6.8 Tris-HCl (Bio-Rad), 26.3% (w/v) glycerol (IBI Scientific, USA), 2.1% SDS (Bio-Rad) and 0.01% bromophenol blue (Sigma). Each protein band was analyzed following previous protocols 14. The blots were incubated with the enhanced chemiluminescence solution (Thermo Fisher Scientific) and detected under a ChemiDoc MP imaging system (Bio-Rad, Hercules, CA, USA). The information of primary and secondary antibodies was shown in Table S1.

### Small interfering RNA (siRNA) silencing

All the siRNAs were synthesized by Integrated DNA Technologies (Coralville, IA). Briefly, the Lipofectamine RNAiMAX transfection reagent (Thermo Fisher Scientific) and the siRNA were diluted in Opti-MEM media respectively and then mixed them for 5 min. After incubation, cells were seeded into plates with transfection mixture simultaneously. The cell viability was assessed by AlamarBlue assay, and the knockdown efficiency was tested by Western blot assay. The sequence information of the siRNAs was listed in Table S2.

### Animal experiments

All experiments applied on animals have been approved by the Animal Research Ethics Committee of the University of Macau and were designed, operated and analyzed in accordance with the ARRIVE (Animal Research: Reporting In Vivo Experiments) guidelines. For both HCT116 and RKO PTEN-isogenic mouse models, *PTEN^+/+^* and *PTEN*^-/-^ cells suspended in Matrigel (Corning) were injected to the two flanks of eight-week-old BALB/c nude mic. Five days after tumor injection, mice were randomly divided into three groups (n = 5 mice per group) and treatment with vehicle (0.9% NaCl, 5% DMSO, 5% tween-80 and 5% PEG-400) or CGM097 at 5 mg/kg, 10 mg/kg or 30 mg/kg dissolved in vehicle buffer via intraperitoneal (I.P.) injection daily for 27 days. Mice weight and tumor volume were measured periodically. Tumor volumes were calculated according to the following modified ellipsoid formula: long axis × short axis^2^ × π/6. Vernier caliper was used to measure the long and short axis of the tumor. At the end of the treatment, mice were sacrificed, and tumors were taken out for weighting and stored in a liquid nitrogen tank.

### Immunofluorescence staining

Cells were seeded into a Nunc Lab-Tek II 8-Chamber Slide (Thermo Fisher Scientific). After treatment, cells were fixed with 4% paraformaldehyde at room temperature for 30 min, and then blocked with 3% BSA in PBS for 30 min. Cells were then incubated with primary antibodies at 4 °C overnight and incubated with secondary antibodies conjugated with Alexa Fluor 488 for 1 h at room temperature in the dark. The cellular nuclei were stained with 1 μg/mL Hoechst 33342 for 10 min. Cells were mounted with Immu-mount (Thermo Fisher Scientific) and observed under a Zeiss LSM 880 Confocal microscope System (Carl Zeiss).

### Reverse transcription-quantitative polymerase chain reaction (RT-qPCR)

All RNA samples were extracted from the cells with Cell Lysates (CL) buffer (10 mM Tris pH7.4 (Invitrogen, USA), 0.25% IGEPAL CA-630 (Sigma), and 150 mM NaCl). Cells were washed by PBS once and lysed with CL buffer for 5 min at room temperature. RNA samples were then instantly performed reverse transcription using High-Capacity cDNA Reverse Transcription Kit (Thermo Fisher Scientific). Purity and concentration of cDNA samples were measured by NanoDrop 2000c (Thermo Scientific). cDNA samples were performed Real Time PCR. ITaq Universal SYBR Green Supermix (Bio-Rad, USA) was used to stain double strand DNA during qPCR at C1000 Touch^TM^ Thermal Cycler (CFX96^TM^ Real-Time System, Bio-Rad). All primers used in this project were synthesized by BGI Genomics (Hong Kong) and the sequences are listed in Table S3.

### Statistical analysis

Statistical significance between control and test groups were determined by Student's t test, one sample t test or two-way ANOVA using Graphpad Prism 6.0. *P* values < 0.05 was considered significant. All the Western blot data shown are representative data from at least three independent experiments.

## Results

### MDM2 inhibition is synthetic lethal with PTEN deficiency in CRC cells

**Figure 1:**
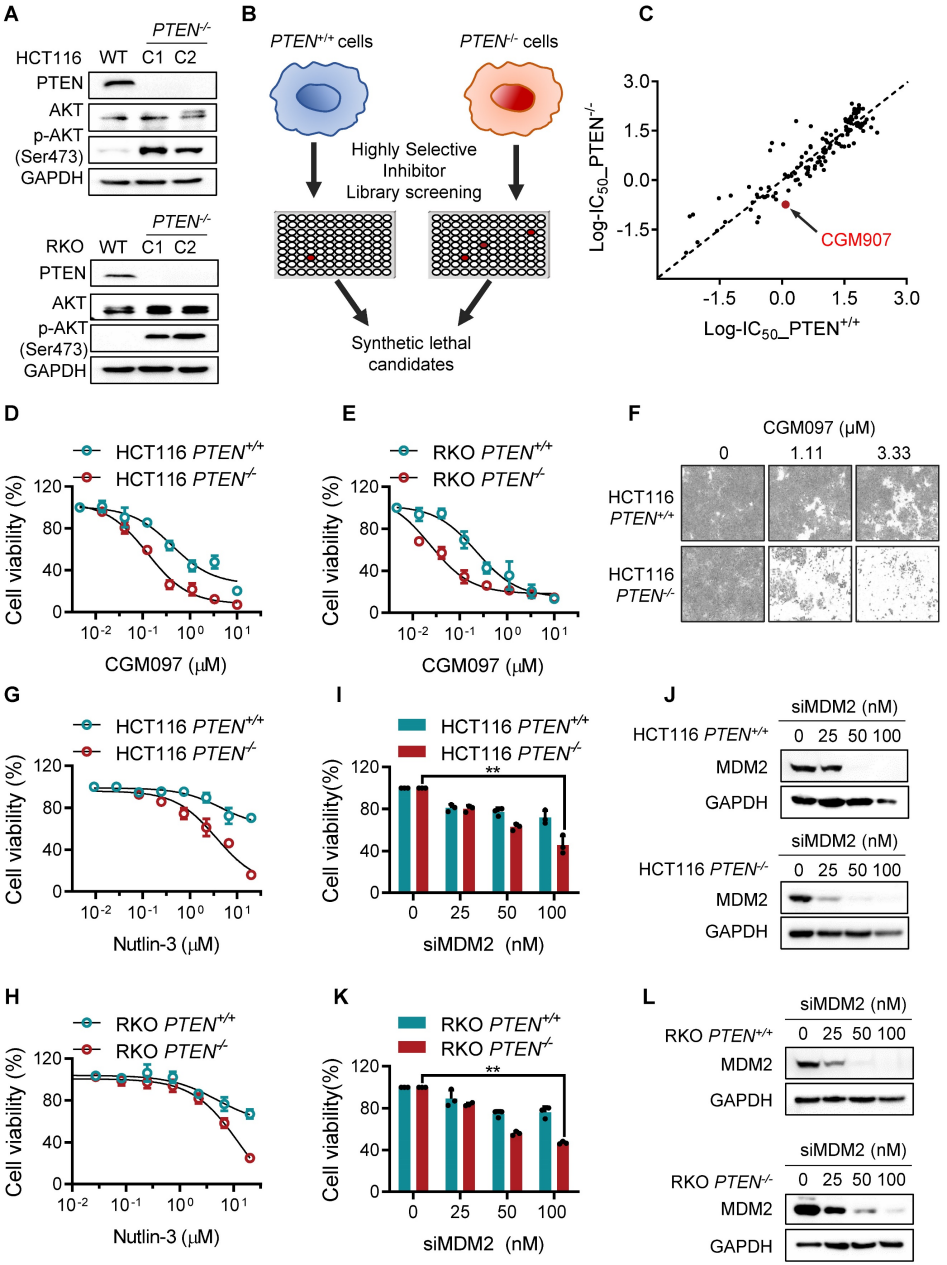
** MDM2 inhibition is synthetic lethal with PTEN deficiency in CRC cells. A.** Western blotting showing no PTEN protein expression in both HCT116 and RKO *PTEN^-/-^* clones. p-AKT was detected for validating PTEN functional deletion. GAPDH, the loading control**. B.** Schematic illustration of the synthetic lethality screenings with the Highly Selective Inhibitor Libraries. **C.** log10-IC_50_ plot of the screening results. A log10 scale of IC50 values of the drugs against HCT116* PTEN^+/+^* and *PTEN^-/-^* cells was plotted. The MDM2 inhibitor is shown with red. **D.** Validation of the synthetic lethality between PTEN and MDM2 using MDM2 inhibitor CGM0979 (**D, E**), Nutlin-3 (**G, J**) and MDM2 siRNA (**H, K**) in HCT116 and RKO PTEN-isogenic cell lines. **F.** The cell images were respective of HCT116 *PTEN^+/+^* and* PTEN^-/-^* cells with CGM097 treatment for 96 h. **I, L** Western blot analysis presented MDM2 knockdown by siRNA in HCT116 and RKO PTEN-isogenic cells. Data are presented as mean ± SD (n = 3 independent experiments), **P < 0.01 between two indicated bars (two-way ANOVA test).

To identify synthetically lethal targets of *PTEN* deficiency in CRC, we generated PTEN-isogenic cell pairs of HCT116 and RKO using CRISPR/Cas 9 gene knockout system. Functional knockout of PTEN in the CRC cells were verified with PTEN status and AKT phosphorylation status in the knockout clones (Figure 1A). The verified PTEN knockout clone (hereafter *PTEN*^-/-^) and the parental HCT116 cell line with wildtype PTEN (hereafter *PTEN*^+/+^) were screened with the Highly Selective Inhibitor Library, containing 318 small molecule inhibitors selectively targeting various cellular druggable targets, to identify synthetic lethal candidates for PTEN deficiency (Figure 1B). As a result, the MDM2 inhibitor CGM097 showed up as one of top candidate drugs that were highly selective toward *PTEN^-/-^* HCT116 cells over *PTEN^+/+^* counterparts (Figure 1C). We then verified the synthetic lethal effect of MDM2 inhibitors on PTEN-deficient CRC cell pairs. We tested two MDM2 inhibitors, including CGM097 and Nutlin-3, which are known to bind to MDM2 and inhibit the interaction between MDM2 and p53, leading to stabilization and activation of p53 15, 16. CGM097 selectively inhibited the growth of *PTEN^-/-^* cells over *PTEN^+/+^* counterparts in both HCT116 and RKO isogenic pairs (Figure 1D-F). Similarly, Nutlin-3 also showed a greater selectivity toward *PTEN^-/-^* CRC cells in both HCT116 and RKO isogenic pairs (Figure G-H), demonstrating that MDM2 inhibitors induce synthetic lethality in PTEN-deficient CRC cells. In addition, siRNA silencing of MDM2 selectively inhibited the growth of *PTEN^-/-^* CRC cells in both HCT116 and RKO (Figure I-L), further verifying synthetic lethal interaction between PTEN and MDM2 in CRC cells in vitro.

### MDM2 inhibitor induces synthetic lethality in PTEN-deficient CRC *in vivo*

**Figure 2:**
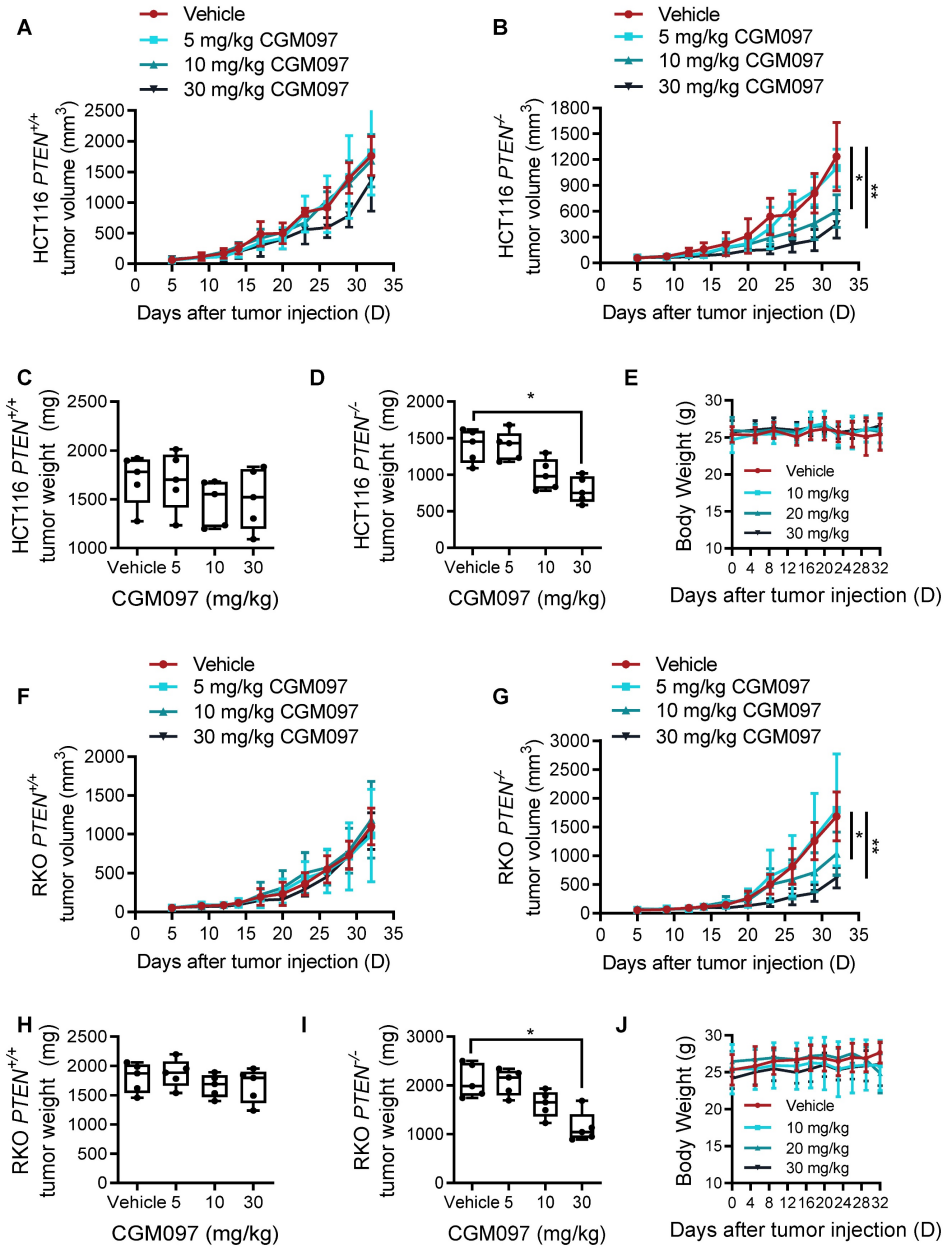
** PTEN and MDM2 show a synthetic lethal interaction in vivo.** Tumor volume (**A, B**) and tumor weight (**C, D**) curves in nude mice bearing HCT116 *PTEN^+/+^* and *PTEN*^-*/-*^ tumor xenografts treated with vehicle, 5 mg/kg CGM097, 10 mg/kg CGM097 or 30 mg/kg CGM097. **E.** HCT116 tumor mice model body weight in nude mice treated by the indicated concentrations of CGM097. Tumor volume (**F, G**) and tumor weight (**H, I**) curves in nude mice bearing RKO *PTEN^+/+^* and *PTEN^-/-^* tumor xenografts treated with the indicated concentrations of CGM097. **K.** RKO tumor mice model body weight in each group were shown. The statistical analysis between two groups in tumor volume and tumor weight was conducted by two-way ANOVA test. *P < 0.05; **P < 0.01 between two indicated bars.

To examine the synthetic lethal effect of MDM2 inhibitor *in vivo*, we conducted tumor xenograft experiments in mice bearing HCT116 *PTEN^+/+^* and *PTEN^-/-^* CRC tumors. Consistent with the in vitro results, intraperitoneal administration of CGM097 dose-dependently inhibited the growth of *PTEN^-/-^* xenograft tumors in mice, while the same dosage regimen did not show meaningful antitumor effect on *PTEN^+/+^* xenograft tumors (Figure 2A-B). The tumor wet weight measurement also showed the selective antitumor effect of CGM097 on *PTEN^-/-^* tumor xenografts (Figure 2C-D). Based on the mouse body weight measurement, the dosage regimen of CGM097 used in this study did not show apparent toxicity (Figure 2E). Moreover, CRC tumor xenograft experiment with RKO PTEN-isogenic pair also showed a similar synthetic lethal effect of CGM097 in PTEN-deficient CRC tumors (Figure 2F-J). These data demonstrate that MDM2 inhibitor induces synthetic lethality in PTEN-deficient CRC in vivo.

### The synthetic lethality of PTEN and MDM2 is p53 dependent

**Figure 3:**
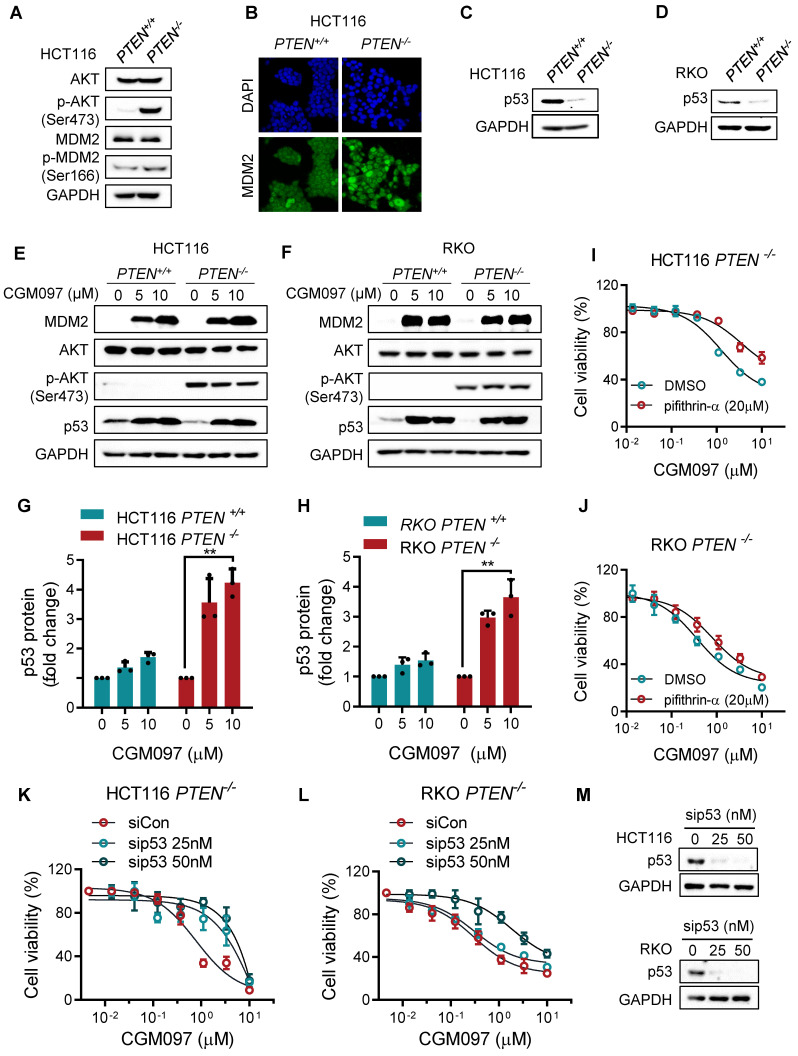
** The synthetic lethality of PTEN and MDM2 is mediated by p53. A.** Immunoblots showing AKT and MDM2 phosphorylation levels in PTEN-isogenic HCT116 cells. **B.** Immunofluorescence analysis of MDM2 cellular distribution, and the nuclei were labeled by DAPI. **C, D.** p53 protein expression levels in HCT116 and RKO PTEN-isogenic cells. Immunoblot showing MDM2, AKT, p-AKT (ser473) and p53 protein expressions with DMSO, 5 μM and 10 μM CGM097 treatment in HCT116 PTEN-isogenic cells (**E**) and RKO PTEN-isogenic cells (**F**). Quantification results of p53 protein expressions in HCT116 (**G**) and RKO (**H**) PTEN-isogenic cells, which were relative to the loading control (GAPDH) and normalized to the corresponding control group. **P < 0.01 between two indicated groups (t test). Rescue experiments with p53 inhibitor pifithrin-α in HCT116 *PTEN^-/-^* cells (**I**) and RKO *PTEN^-/-^* cells (**J**). The cells were cotreated with pifithrin-α and CGM097 for 72 h, and the cell viability was measured by AlamarBlue assay. **K-L.** HCT116 and RKO *PTEN^-/-^* cells were transfected with p53 siRNA and co-incubated with CGM097 for 72 h. The cell viability was measured as the same method above.** M.** Immunoblots indicating p53 siRNA transfection efficiency.

**Figure 4:**
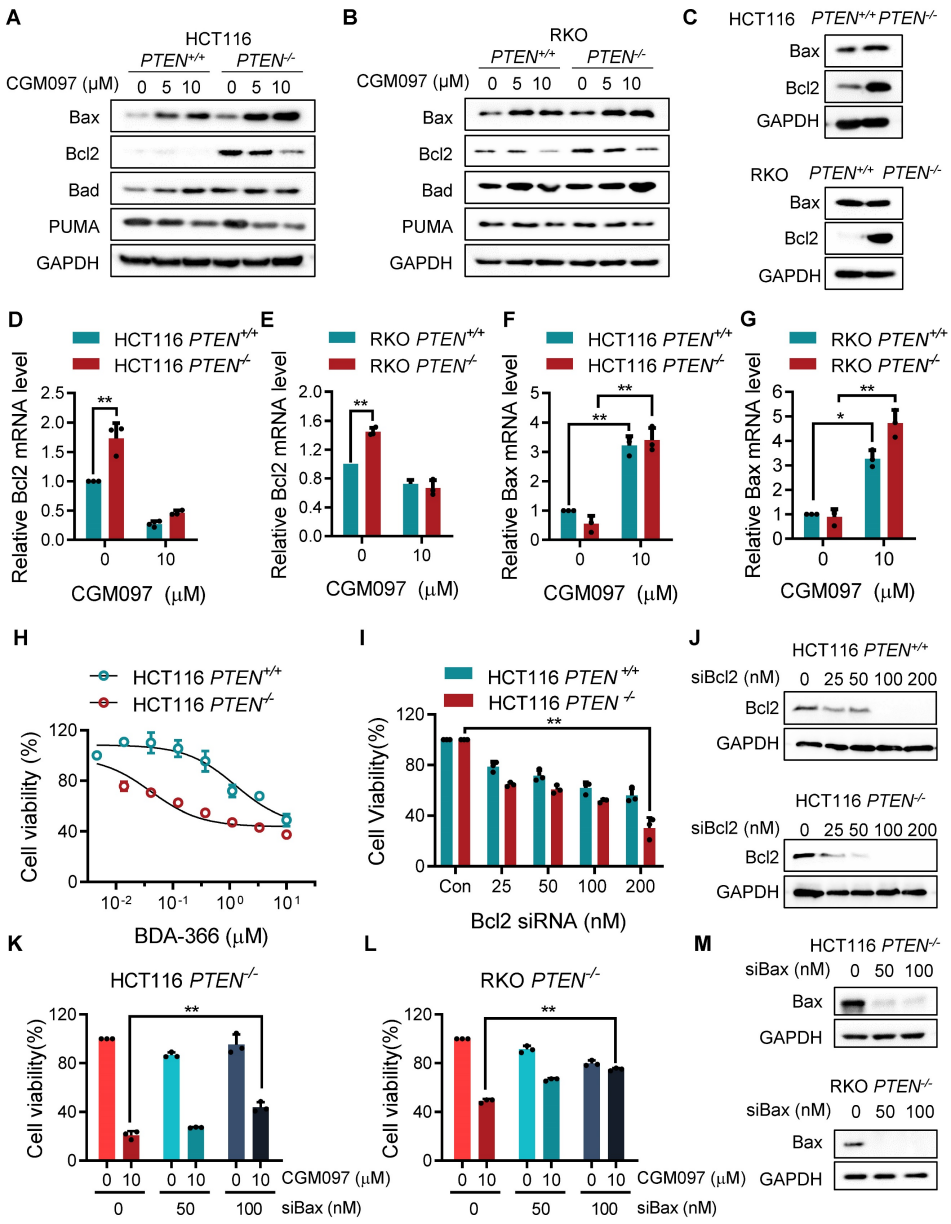
** MDM2 inhibition modulates Bcl2/Bax ratio in PTEN-deficient CRC cells.** Apoptosis related protein expressions in HCT116 PTEN-isogenic pairs (**A**) and RKO PTEN-isogenic pairs (**B**). **C.** Bax and Bcl2 proteins in HCT116 and RKO PTEN-isogenic cells were tested via immunoblots. qRT-PCR was performed to evaluate mRNA levels of Bcl2 (**D, F**), and Bax (**E, G**) in HCT116/RKO* PTEN^+/+^* and *PTEN^-/-^* cells. Cell viability of HCT116* PTEN^+/+^* and *PTEN^-/-^* cells after culturing with Bcl2 inhibitor BDA-366 (**H**) or Bcl2 siRNA (**I**). Rescue assay showing the effect of Bax knockdown on MDM2 inhibition in HCT116 *PTEN^-/-^* cells (**K**) and RKO* PTEN^-/-^* cells (**L**). **J, M.** Bcl2 and Bax siRNA transfection efficiency were shown by immunoblots. Data are presented as mean ± SD (n = 3 independent experiments), *P < 0.05; **P < 0.01 between two indicated bars (two-way ANOVA test).

To explore mechanistic insight in the synthetic lethal interaction between PTEN and MDM2, we first analyzed the relationship between PTEN and MDM2 signaling pathways. There was no meaningful correlation between total PTEN and MDM2 protein levels (Supplementary Figure S1). However, *PTEN^-/-^* cells showed a high level of phosphorylated AKT, and MDM2 phosphorylation and nuclear localization were increased in *PTEN^-/-^* cells compared to *PTEN^+/+^* counterparts (Figure 3A-B), indicating that MDM2 is activated in PTEN-deficient CRC cells. This result was consistent with previous reports that PTEN restricted MDM2 in cytoplasm by suppressing AKT activity, thereby neutralizing the MDM2 inhibition effect on p53 17. AKT is known to phosphorylate MDM2 at Ser166 or 186, promote MDM2 nuclear translocation and enhance MDM2-mediated p53 destabilization 18, 19. Indeed, p53 protein level was significantly reduced in *PTEN^-/-^* HCT116 or RKO cells (Figure 3C-D). We then analyzed the MDM2 and p53 levels in PTEN-isogenic CRC cells treated with the MDM2 inhibitor CGM097. CGM097 strongly increased MDM2 and p53 levels in both *PTEN^-/-^* and *PTEN^+/+^* CRC cells (Figure 3E-F). It has been expected that p53 level would be increased by MDM2 inhibitor, and MDM2 itself as a transcription target of p53 would be increased as well. The level of AKT or phosphorylated AKT was not changed by the MDM2 inhibitor. Of note, despite similar level of increase in p53 by the MDM2 inhibitor in both* PTEN^-/-^* and *PTEN^+/+^* CRC cells, the fold increase in the p53 level to its basal level was significantly higher in *PTEN^-/-^* CRC cells (Figure 3G-H). These results suggested that *PTEN^-/-^* CRC cells could have received a much bigger impact by p53 activation than *PTEN^+/+^* cells. Therefore, to test the possibility that p53 activation is the key factor mediating the differential sensitivity of *PTEN^-/-^* and *PTEN^+/+^* CRC cells to MDM2 inhibitor, we conducted p53 phenotype rescue experiments with a siRNA and a small molecule inhibitor of p53. The p53 inhibitor pifithrin-α partially reversed the inhibitory effect of MDM2 inhibitor on the growth of *PTEN^-/-^* CRC cells (Figure 3I, J). Furthermore, the depletion of p53 using siRNA significantly reversed MDM2 inhibitor IC_50_ by 5-fold (from 0.99 μM to 5.20 μM) in *PTEN^-/-^* CRC cells (Figure 4K-M). Of note, the rescue effect was much stronger with p53 siRNA than pifithrin-α. This was presumably because pifithrin-α inhibits only a part of p53 transcription targets 20, hence showing less inhibitory effects on p53 than that by siRNA silencing. In addition, MDM2 inhibitor did not elicit synthetic lethal effect on PTEN-isogenic DLD cells where p53 is mutated (Supplementary Figure S2). Collectively, these results indicated that PTEN-deficient CRC cells harbored a low basal level of p53 due to the activated AKT-MDM2 axis, while a remarkable activation of p53 in PTEN-deficient CRC cells upon MDM2 inhibition could trigger the selective antitumor effect in the cells.

### MDM2 inhibition reverses Bcl2/Bax ratio in PTEN-deficient CRC cells

**Figure 5:**
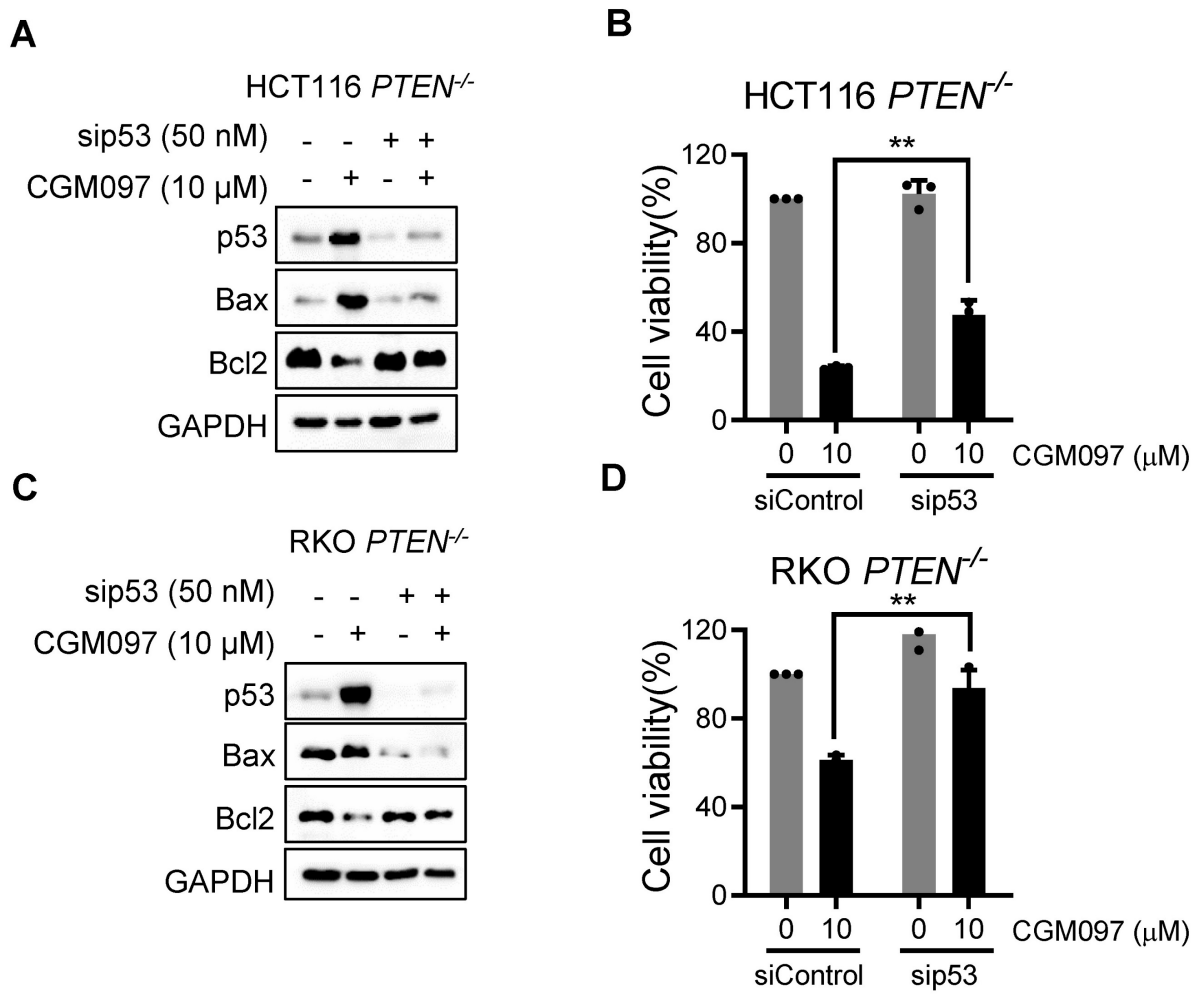
** p53 governs MDM2 inhibitor effects on Bcl2/Bax ratio and PTEN-deficient CRC cell death. A, C** Immunoblot showing Bax and Bcl2 protein expression in HCT116 and RKO *PTEN^-/-^* cells upon p53 silencing with or without MDM2 inhibitor (CGM097) treatment. **B, D.** Viability analysis in p53 knockdown HCT116 and RKO *PTEN^-/-^* cells after CGM097 treatment. Data are presented as mean ± SD (n = 3 independent experiments), **P < 0.01 between two indicated bars (t test).

In this study, we showed that PTEN-deficient CRC cells exhibited much higher fold-increase in p53 level upon MDM2 inhibitor treatment, and such a differential activation of p53 could provide the differential sensitivity of CRC cells to MDM2 inhibitor. To further delineate the role of p53 on the synthetic lethal effect, we analyzed p53 downstream effector targets that execute cell growth inhibition and apoptosis. Among the p53 effector targets we analyzed, the change in the levels of Bad and PUMA did not show meaningful difference between the two PTEN-isogenic CRC cell lines (Figure 4A-B). However, the Bcl2 basal level was significantly higher in *PTEN^-/-^* CRC cells (Figure 4C) and was dose-dependently reduced by the MDM2 inhibitor (Figure 4A-B). Bax level was dose-dependently increased by the MDM2 inhibitor in both *PTEN^-/-^* and *PTEN^+/+^* CRC cells. It was assumed that p53 could be the upstream transcription factor that regulated the expression of Bax and Bcl2. We then analyzed the mRNA levels of Bcl2 and Bax in PTEN-isogenic cell lines treated with the MDM2 inhibitor. The basal mRNA level of Bcl2 was significantly higher in *PTEN^-/-^* CRC than that in *PTEN^+/+^* CRC cells (Figure 4D-E). MDM2 inhibition strongly reduced the level of Bcl2 mRNA in both *PTEN^-/-^* and *PTEN^+/+^* CRC cells. Bax basal mRNA level was similar in *PTEN^-/-^* and *PTEN^+/+^* CRC cells, and inhibition of MDM2 significantly increased Bax mRNA in both cell lines (Figure 4F-G). These results implied that the reduced p53 basal level in *PTEN^-/-^* CRC cells led to an increase in Bcl2 basal transcripts in the cells since p53 acts as a transcription repressor of Bcl2. There was no difference in Bcl2 protein stability between *PTEN^-/-^* and *PTEN^+/+^* CRC cells, further supporting the notion of Bcl2 transcription regulation by p53 (Supplementary Figure S3). The low basal of p53 and the high basal level of Bcl2 could contribute to the cellular oncogene addiction to this pathway for cell survival and anti-apoptotic phenotypes. MDM2 inhibition strongly increased p53 level, thereby reversing Bcl2/Bax ratio and triggering pro-apoptotic phenotype in *PTEN^-/-^* CRC cells. To examine the role of the Bcl2/Bax ratio change in the synthetic lethal phenotype of *PTEN^-/-^* CRC cells, we first used a Bcl2 inhibitor and siRNA on the cell viability in the PTEN-isogenic cell pair. Treatment of cells with the small molecule Bcl2 inhibitor BDA-366 or Bcl2 depletion using siRNA was able to induce synthetic lethal phenotype in *PTEN^-/-^* CRC cells (Figure 4H-J). This result supported the idea of the Bcl2 oncogene addiction in PTEN-deficient CRC cells. Next, the depletion of Bax using a siRNA dose-dependently rescued the cell death induced by the MDM2 inhibitor in *PTEN^-/-^* CRC cells (Figure 4K-M). These results suggested that p53 reduction in PTEN-deficient CRC cells led to the Bcl2 oncogene addiction state and activation of p53 by MDM2 inhibitor completely shifted the Bcl2/Bax ratio, inducing apoptotic cell death in the cells. To further prove this model, we examined the effect of p53 depletion on MDM2 inhibitor-induced Bcl2/Bax ratio change and cell death in *PTEN^-/-^* CRC cells. CGM097 treatment significantly increased p53 level, followed by the increase in Bax and the reduction in Bcl2 levels *PTEN^-/-^* HCT116 cells (Figure 5A). The depletion of p53 by the siRNA completely reversed the Bcl2/Bax ratio and significantly rescued the cell death in *PTEN^-/-^* HCT116 cells treated with the MDM2 inhibitor (Figure 5B). A similar result was observed in *PTEN^-/-^* RKO cells treated with the MDM2 inhibitor (Figure 5C-D), supporting our proposed model.

### MDM2 inhibition selectively induced mitochondria-mediated apoptosis in PTEN-deficient CRC cells

**Figure 6:**
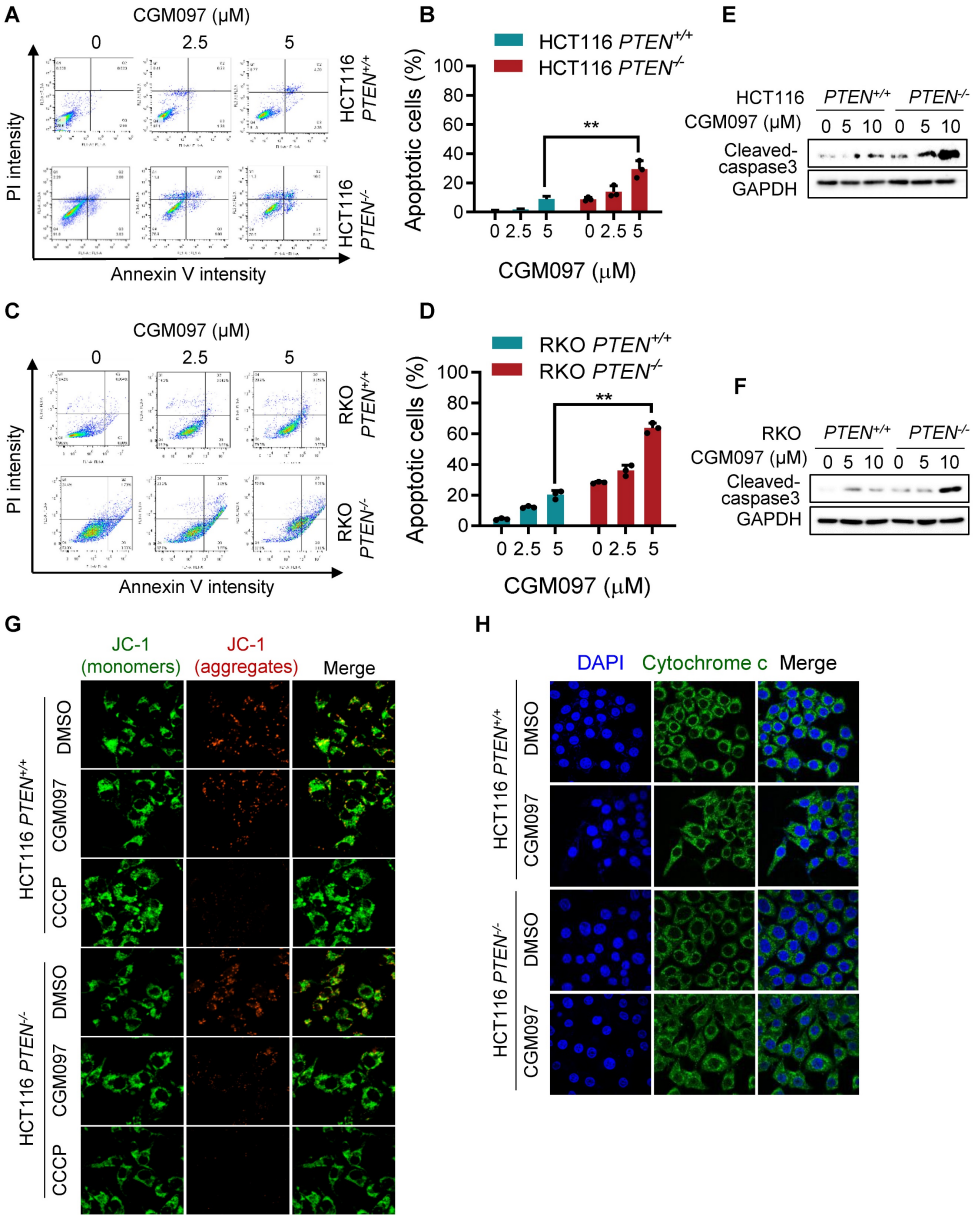
** MDM2 inhibition induces mitochondria-mediated apoptosis in PTEN-deficient CRC cells.** HCT116 (**A**) and RKO (**C**) PTEN-isogenic cells were treated with MDM2 inhibitor (CGM097) for 72 h, then labelled with Annexin V-Alexa Flour 488 (AF488) and propidium iodide. The flow cytometry data (**A, C**) and apoptotic cells quantitative data (**B, D**) are shown. All data were represented as mean± SD, *P < 0.01. **E, F.** Western blot analysis represented cleaved caspase 3 in HCT116 (**E**) and RKO (**F**) PTEN-isogenic cells in respond to CGM097 for 48 h. **G.** The mitochondrial membrane potential of HCT116 PTEN-isogenic cells were measured by JC1 staining after CGM097 treatment. CCCP served as a positive control for reduction in mitochondrial membrane potential. **H**. Effect of CGM097 on cellular distribution of cytochrome c (green) in HCT116 *PTEN^-/-^* cells. The nucleuses were labelled with DAPI (blue).

We showed that MDM2 inhibition induced p53-mediated reversal of Bcl2/Bax ratio and led to a selective cell death in PTEN-deficient CRC cells. It is well recognized that the pro-apoptotic protein Bax promotes apoptotic cell death by facilitating mitochondrial outer membrane permeabilization (MOMP), causing cytochrome c release and apoptosome-mediated intrinsic apoptosis 21. Bcl2 antagonizes this process by binding to Bax 22. To determine the MDM2 inhibitor-induced cell death phenotype, we first analyzed apoptotic cell staining with Annexin V and propidium iodide. Flow cytometry analysis showed that the MDM2 inhibitor significantly induced apoptosis in *PTEN^-/-^* CRC cells in a dose-dependent manner (Figure 6A-D), with a marginal effect on *PTEN^+/+^* CRC cells. We also observed the selective caspase-3 activation in* PTEN^-/-^* CRC cells by the MDM2 inhibitor (Figure 6E-F). We next measured realtime MOMP induction in cells by the MDM2 inhibitor using JC-1 fluorescent dye. The JC-1 is a cationic dye that accumulates in mitochondria depending on mitochondrial membrane potential. It forms J-aggregates yielding red fluorescence at a high membrane potential and exists as a monomer yielding green fluorescence at a low membrane potential, thus enabling to quantitate MOMP. CGM097 did not induce measurable MOMP in *PTEN^+/+^* CRC, while it significantly induced MOMP in *PTEN^-/-^* CRC (Figure 6G). CCCP, an uncoupler of mitochondrial oxidative phosphorylation, was used as a positive control compound for MOMP. Lastly, we analyzed mitochondrial cytochrome c release upon MOMP induced by MDM2 inhibitor. Immunofluorescence staining of cytochrome c showed that CGM097 selectively induced cytochrome c release from mitochondria in *PTEN^-/-^* CRC cells, which was indicated by a diffused staining pattern of cytochrome c throughout the cytoplasm (Figure 6H). Cytochrome c in *PTEN^+/+^* CRC treated with CGM097 largely restrained in the mitochondria. These results demonstrated that MDM2 inhibition selectively induced mitochondria-mediated apoptosis, accompanying MOMP, in PTEN-deficient CRC cells.

## Discussion

**Figure 7:**
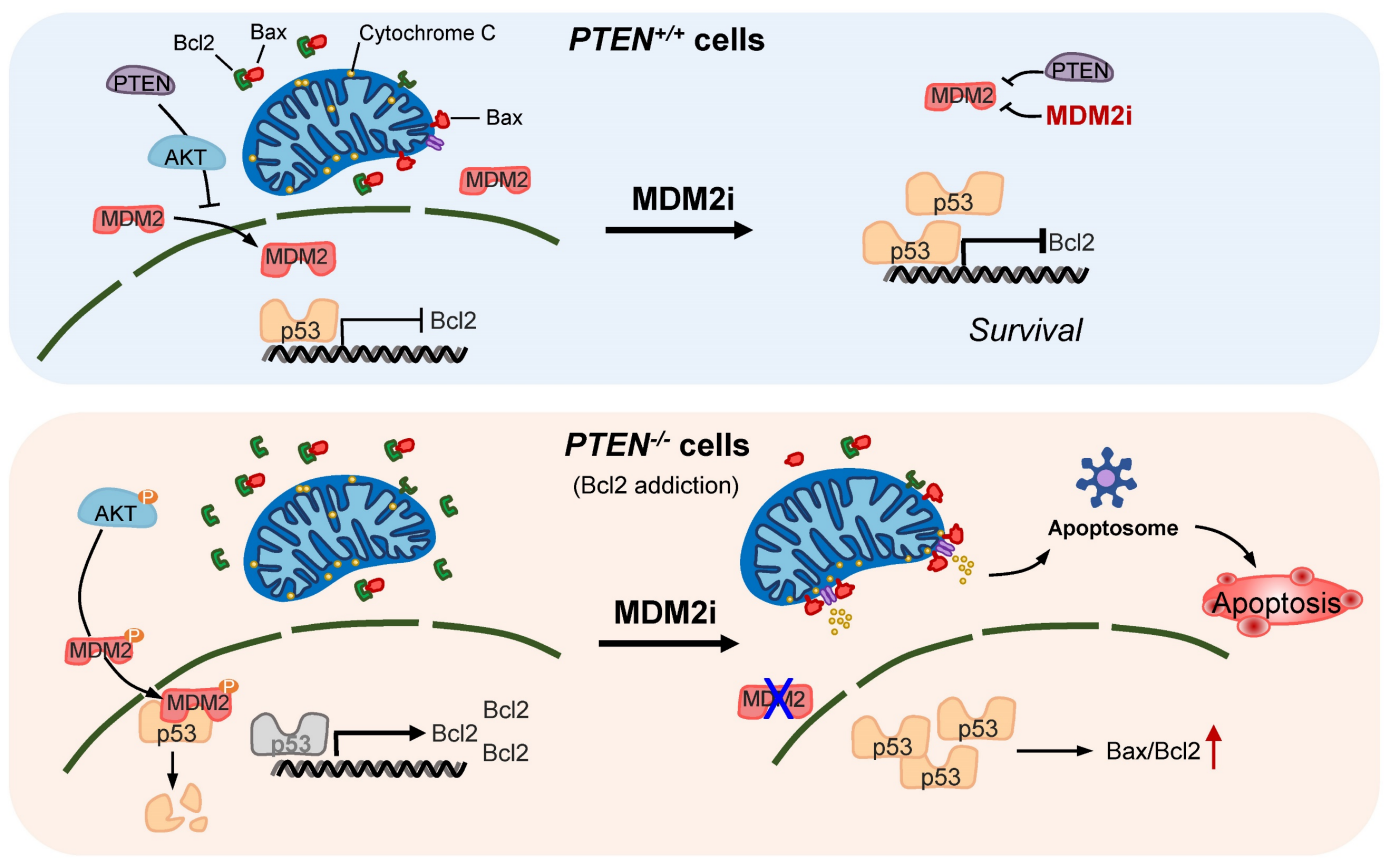
Proposed working model of the synthetic lethality between PTEN and MDM2. In PTEN-wildtype cells, PTEN suppresses AKT activity, thereby limiting MDM2's inhibitory effect on p53. p53 in turn controls Bcl2 transcription. Inhibition of MDM2 in these cells has limited effect on p53-Bcl2 pathway. In PTEN-deficient cells, active AKT promotes MDM2 activity, thereby reducing p53 basal level and inhibiting p53's transactivation function. Bcl2 transcription is de-repressed and its expression is highly elevated in these cells, making the cells addicted to Bcl2. Inhibition of MDM2 in these cells activates p53, shifts Bax/Bcl2 ratio and induces mitochondria-mediated apoptosis.

PTEN loss or mutational inactivation is one of key drivers in CRC progression and metastasis with hyper-activation of PI3K/AKT pathway. Furthermore, PTEN loss is known to impair the efficacy of the receptor tyrosine kinase (RTK)-targeted therapies, such as cetuximab and trastuzumab in the clinic 8, 23. Despite the critical role of PTEN loss in CRC progression and therapy resistance, effective therapeutic strategy targeting PTEN loss has not been introduced in the clinic 24. In this study, we report that MDM2 inhibition induces synthetic lethality in PTEN-deficient CRC cells. Small molecule MDM2 inhibitors or MDM2 depletion by siRNA selectively inhibited the growth of PTEN-deficient CRC cells. The synthetic lethal effect of the MDM2 inhibitor was further validated in mice tumor xenograft models of PTEN-isogenic CRC. Our in-depth mechanistic study of the synthetic lethality revealed the following model: (1) PTEN loss leads to a reduced p53 basal level following AKT-MDM2 axis activation. (2) Reduced p53 level in turn elevated the expression of Bcl2, causing the cellular Bcl2 addiction in PTEN-deficient CRC cells. (3) Inhibition of MDM2 leads to an activation of p53, significantly reversing the Bcl2/Bax ratio in PTEN-deficient CRC cells. (4) The reversal of Bcl2/Bax ratio leads to the induction of MOMP, followed by the release of cytochrome c and mitochondria-mediated apoptotic cell death in PTEN-deficient CRC cells. (5) PTEN-proficient CRC cells have a normal level of p53 and a balanced Bcl2/Bax ratio, hence showing resistance to MDM2 inhibitor effects (summarized in Figure 7).

PTEN and p53 are the most frequently mutated tumor suppressors in human cancers. With their essential roles in tumor suppression, the two proteins functionally interact with each other and cooperatively participate in the suppression of tumor initiation and development. p53, as a transcription factor, binds to a p53 binding element on PTEN gene promoter and activates the transcription of PTEN 25. Loss of p53 decreases PTEN expression and enhances PI3K/AKT pathways leading to activation of oncogenic transcription factors 26. On the other hand, PTEN promotes p53 activity by negatively regulating the activity of the p53 inhibitor MDM2. MDM2 is a negative regulator of p53 by binding to p53 and inhibiting p53 transcription activity or promoting p53 degradation with its E3 ubiquitin ligase activity 13. PI3K/AKT pathway promotes MDM2 nuclear translocation by phosphorylating Ser166 and Ser186 of MDM2, which enhances MDM2-mediated ubiquitination and degradation of p53 19, 27. Restoring PTEN expression in PTEN-null glioblastoma cells protects p53 from MDM2-mediated degradation and sensitizes cancer cells to chemotherapy 17. Our study showed a consistent result with these findings that MDM2 phosphorylation and nuclear localization were significantly increased in PTEN-deficient cells where AKT was highly activated. Concomitantly, p53 basal level was significantly lower in PTEN-deficient CRC cells than in PTEN-proficient counterparts. These data suggest that PTEN-deficient CRC cells have adapted under a cell survival condition where MDM2 is active and p53 is suppressed. Inhibition of MDM2 induced synthetic lethality in PTEN-deficient CRC cells with a dramatic increase in the level of p53 in the cells, suggesting that activation of p53 in the p53-suppressed cells triggered p53-mediated apoptotic cell death. The synthetic lethal effect of MDM2 inhibitor on PTEN-deficient CRC cells was largely abrogated in the absence of p53, further verifying this notion.

More recent studies have shown that MDM2 could facilitate NEDD8 conjunction of p53, resulting in neddylation of p53, which was independent of MDM2 ubiquitin ligase function. NEDD8 is one of ubiquitin-like proteins, which displays similar ubiquitination function of MDM2. MDM2-mediated NEDD8 conjugation of p53 results in transcriptionally inactive p53 28. Further studies showed that constitutively active growth factor signaling or its downstream c-Src switches MDM2 function to a neddylating enzyme, whereas MDM2 plays a role as a ubiquitin ligase in response to DNA damage stress 29. In our study, MDM2 phosphorylation was increased upon the activation of AKT in PTEN-deficient CRC cells and CGM097 treatment significantly increased the level of MDM2 as an auto-regulatory feedback mechanism upon p53 activation. The increase in the MDM2 level could potentially counteract p53-induced apoptosis and may reduce the antitumor efficacy of the drug, which was shown as incomplete tumor cell death by CGM097 in our in vitro and in vivo studies. Since DNA damage stress has not been observed upon CGM097 treatment (data not shown), this notion suggests that the elevation of MDM2 level upon CGM097 treatment could undermine the effect of the drug-induced apoptosis, possibly by neddylating p53 or using alternative MDM2-mediated survival pathways independent of p53. MDM2 is known to exert multiple oncogenic functions independent of p53. MDM2 targets the tumor suppressive transcription factor FOXO3a for ubiquitin-proteasome dependent degradation via RAS/ERK pathway 30. Other tumor suppressors, including E-cadherin 31 and the retinoblastoma protein (RB1) 32 are MDM2 substrates for degradation. In contrast, MDM2 binds to the oncogenic transcription factors E2F1/DP1 and stimulates their transcriptional activity to positively augment cell proliferation 33. Therefore, the development of strategies to minimize the feedback loop of the MDM2 expression by MDM2 inhibitors is necessary to improve the antitumor efficacy.

Bcl-2 family proteins are central regulators of apoptosis. They can be classified into three classes, including anti-apoptotic Bcl2 family (Bcl2, Bcl-XL, Mcl1, etc), pro-apoptotic Bcl2 family (Bax, Bak and Bok) and BH3-only proteins (Bad, Bik, Bid, PUMA, etc). By regulating the integrity of mitochondrial outer membrane, Bcl2 family proteins dictate the cell fate decision between cell survival and death. Among the Bcl2 family proteins, Bcl2 and Bax are the most well characterized members and are representative members of anti-apoptotic and pro-apoptotic proteins, respectively. Based on the classic model, soluble Bax proteins undergo a conformational change upon apoptotic signal and are oligomerized at the mitochondrial outer membrane to form a channel. This channel mediates the release of proteins, such as cytochrome c and other soluble proteins, into the cytosol. This process is called mitochondrial outer membrane permeabilization (MOMP) 34, 35. The release of cytochrome c into cytosol induces the formation of the apoptosome and triggers caspase-mediated apoptosis 36. Bcl2, as an anti-apoptotic member, directly binds to Bax and inhibits Bax-induced, mitochondria-mediated apoptosis 37. As exemplified with Bax and Bcl2, interactions between these counteracting BCL-2 family proteins determine the cell fate via a balance between anti-apoptotic and pro-apoptotic proteins 38. Overexpression of Bcl2 is common in many types of human cancer and is highly correlated with therapy resistance. In general, Bcl2 overexpression does not promote cell proliferation, but rather, overexpression of Bcl2 inhibits cell death via evading apoptotic checkpoint 39. Cancer cells with a high level of Bcl2 have unbalanced apoptotic regulators and are addicted to Bcl2. These cells are highly dependent on Bcl2 for survival and hence are vulnerable to the inhibitors of Bcl2, making it an attractive therapeutic target 40.

Both Bcl2 and Bax are transcription targets of p53. p53 is a well-known transcription activator for Bax for apoptosis induction 41, while p53 acts as a transcription repressor of Bcl2 by binding to p53-negative response element on Bcl2 promoter 42. p53 is also known to directly bind and antagonize Bcl2 function independently of its transactivation function 43. Therefore, p53-mediated regulation of the ratio of Bax versus Bcl-2 protein level can influence cell fate 44. Our study shows that Bcl2 level is highly elevated in PTEN-deficient CRC cells, causing the cellular addiction to Bcl2. MDM2 inhibition significantly increased p53 level, followed by the reduction of Bcl2 and elevation of Bax levels in PTEN-deficient CRC cells. Depletion of p53 in these cells reversed the Bcl2/Bax ratio, leading to the rescue of the synthetic lethal effect of MDM2 inhibitor. We lastly demonstrated that MDM2 inhibitor induced the mitochondria-mediated apoptosis, accompanying MOMP and cytochrome c release, strongly supporting our mechanistic model of the synthetic lethality.

In conclusion, this study shows that MDM2 inhibition is synthetic lethal with PTEN loss in CRC cells. In vivo validation and further mechanistic deconvolution of the synthetic lethality suggest that pharmacological inhibition of MDM2 or Bcl2 could be a potential therapeutic strategy for the treatment of CRC with PTEN loss.

## Supplementary Material

Supplementary figures and tables.
