# Supplementary material for: MDM2 inhibition is synthetic lethal with PTEN loss in colorectal cancer cells via the p53-dependent mechanism
Source: Int J Biol Sci. 2023 Jul 9;19(11):3544–57. doi: 10.7150/ijbs.82566 (PMC10367566; doi:10.7150/ijbs.82566)
Supplement: Supplementary file 1 — Supplementary figures and tables. [file ijbsv19p3544s1.pdf]

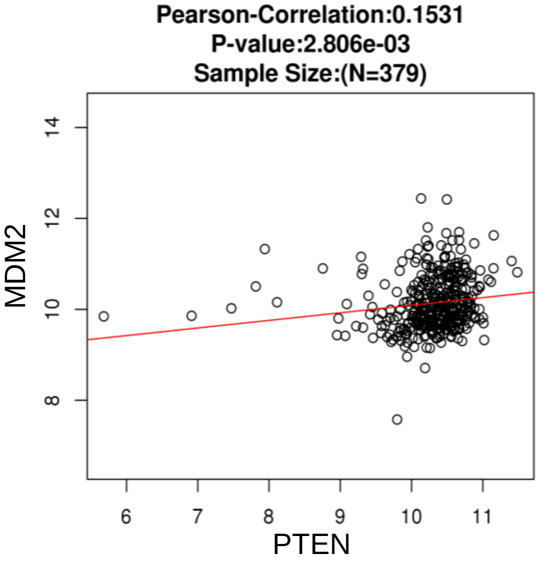

Figure S1. The relationship between PTEN mRNA and MDM2 mRNA levels was analyzed in colorectal adenocarcinoma patients from TCGA database with LinkedOmics (http://www.linkedomics.org). The Pearson correlation coefficient of 0.1531 and the P value of 0.002 were shown from the analysis.

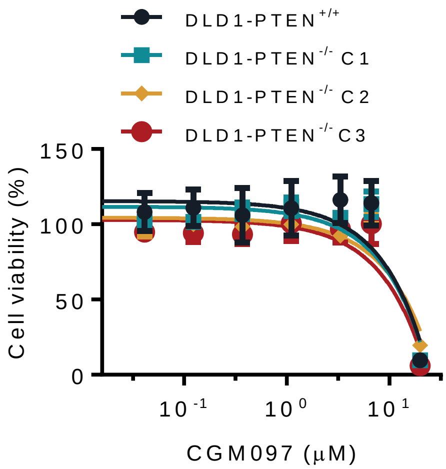

Figure S2. Cell viability analysis of DLD1 wild type cells and three DLD1 PTEN knock out clones with CGM097 treatment.

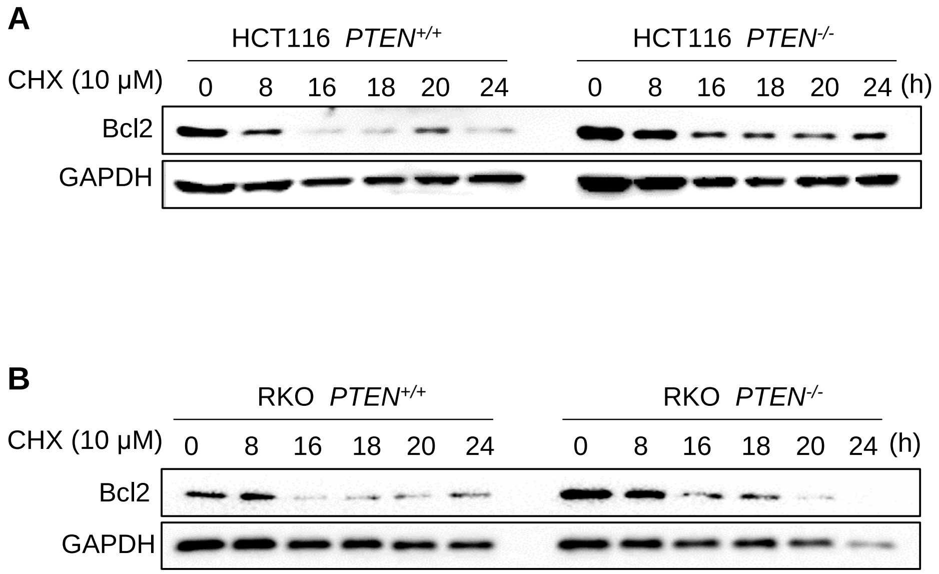

Figure S3. Effect of PTEN deficiency on Bcl2 protein stability A and B, Bcl2 protein stability was examined with cycloheximide-chase assay and Western blots in HCT116 (A) and RKO (B) PTEN-isogenic cells. Bcl2 half-life is similar between PTEN-wildtype and PTEN-deficient CRC cells.

Supplementary Table 1. Antibodies use in this study.

No	Primary Antibodies	Suppliers	Catalog No.	Molecular weight (kDa)
1	PTEN (A2B1)	Santa Cruz Biotechnology	sc-7974	55
2	AKT (B1)	Santa Cruz Biotechnology	sc-5298	62
3	p-AKT (ser473)	Cell Signaling Technology	9271	60
4	GAPDH	Santa Cruz Biotechnology	sc-365062	37
5	MDM2 (D1V2Z)	Cell Signaling Technology	86934	90
6	p-MDM2 (ser166)	Cell Signaling Technology	3521	90
7	Cleaved Caspase-3 (Asp175)	Cell Signaling Technology	9661S	17, 19
8	P53 (DO-1)	Santa Cruz Biotechnology	sc-126	53
9	Bax (B-9)	Santa Cruz Biotechnology	Sc-7480	23
10	Bcl2 (C-2)	Santa Cruz Biotechnology	Sc-7382	26
11	Bad	Cell Signaling Technology	9292	23
12	Puma (E2P7G)	Cell Signaling Technology	98672	23
13	Phospho-p53 (Ser46)	Beyotime	AF5896	44
14	Histone H2A.X	Cell Signaling Technology	7631	15

Supplementary Table 2. Sequences of siRNAs used in this study

No	Target gene	Suppliers	Sequence
1	MDM2	Integrated DNA Technologies	5’-GCAACUUUACUAAUGGUA-3’
2	Bcl2	Integrated DNA Technologies	5’-UGAUGAGAUACAUCCAUU-3’
3	Bax	Integrated DNA Technologies	5’-GAGUGGCAGCUGACAUG-3’
4	p53	Integrated DNA Technologies	5’-UCAAAUCAUCCAUUGCUUGGG-3’

Supplementary Table 3. Sequences of qPCR primer pairs used in this study.

No	Target gene	Suppliers	Sequence
1	Bax	BGI	Forward (5'-CCCGAGAGGTCTTTTTCCGAG-3’)<br>Reverse (5'-CCAGCCCATGATGGTTCTGAT-3’)
2	Bcl2	BGI	Forward (5'-GGTGGGGTCATGTGTGTGG-3’)<br>Reverse (5'-CGGTTCAGGTACTCAGTCATCC-3’)
3	GAPDH	BGI	Forward (5'-GTGGACCTGACCTGCCGTCT-3’)<br>Reverse (5'-GGAGGAGTGGGTGTCGCTGT-3’)
